# Pain Prevalence in Multiple Sclerosis in a Lisbon Tertiary Hospital: A Cross-Sectional Study

**DOI:** 10.7759/cureus.22213

**Published:** 2022-02-14

**Authors:** Ana Filipa Junqueira, Sílvia Farraposo, Ana Rita Cruz, Mónica Paes Mamede, Leonor Silva, Graça Mesquita

**Affiliations:** 1 Anesthesiology, Centro Hospitalar Universitário de Lisboa Central, Lisboa, PRT

**Keywords:** anesthesia, acute pain, chronic pain management, heath related quality of life, multiple sclerosis

## Abstract

Background: Multiple sclerosis is a chronic neurological disease with increasing incidence and prevalence worldwide being the main cause of non-traumatic disability in young adults. Both acute and chronic pain have been mentioned as the most common symptoms among those patients.

Objective: This study was designed to evaluate the pain experience among patients with multiple sclerosis by describing its prevalence, characteristics, analgesic treatment and its efficacy, and also the impact of pain on quality of life.

Methods: A cross-sectional observation survey was carried out on patients with multiple sclerosis followed in a tertiary hospital. Data were collected between December 2019 and March 2021 from a structured telephone inquiry, applying two questionnaires, the Brief Pain Inventory and the McGill Pain Questionnaire (MPQ), to evaluate the prevalence of pain and its impact on quality of life (QoL). Clinical records were also consulted to obtain data on disease duration, year of diagnosis, MS type, Expanded Disability Status Scale (EDSS) score.

Results: Our sample included 305 patients in a universe of 1500, mainly women, with mean age of 44.27 years, and most of them presented with an outbreak-remission subtype of disease. One hundred twenty-four patients experienced pain which corresponds to 41% of the patients. Considering the patients who experienced pain, 67.7% were under treatment and of these, 64.3% with only one painkiller. Pain significantly interfered with general activity, mood, and regular work.

Conclusion: Pain was an important symptom in this group of patients with MS and significantly interfered with mood, general activity, and regular work. The maximum intensity of pain felt by patients was significant and only 67.7% of patients were under analgesic treatment with mean pain relief of 54. NSAIDs were the most used drugs followed by gabapentinoids and acetaminophen for the management of pain. Medical community must continue to study this population in order to improve the approach to pain in these patients and improve quality of life.

## Introduction

Multiple sclerosis (MS) is an autoimmune disorder of the central nervous system (CNS) that is characterized by patchy inflammation, gliosis, and demyelination within the central nervous system. Its increasing incidence and prevalence worldwide make it the main cause of non-traumatic disability in young adults [1]. It is usually diagnosed around the third decade of life and is more prevalent in females [1,2].

The severity of the disease depends on its form, duration, location, and size of lesions in the CNS, which are highly variable. Several forms of the disease can be described like relapsing-remitting MS (RRMS), secondary progressive MS (PSMS), and primary progressive MS (PPMS). Common symptoms include sensory impairment, extremity weakness, urinary dysfunction, visual impairment, fatigue, spasticity, incoordination, and pain [3,4].

Pain is a frequent complaint among individuals with MS. The prevalence of pain in MS differs in the literature, ranging from 28% to 92% [5,6]. This variation is due to methodological differences across studies regarding the patient source, method of sampling, research design, heterogeneity and complexity of the disease itself, and pain measurement. The mechanisms of pain are poorly understood [6,7]. MS pain may be mixed and divided into two different types - neuropathic and nociceptive. Neuropathic pain may be a consequence of damage to myelinated nerves in CNS, which often causes sudden and sharp pain. The sensation can be lightning-like and intermittent, or it can be a burning, tingling, or a tight “hug-like” feeling that can be continuous. On the other hand, nociceptive pain arises from activation of peripheral nociceptors due to tissue damage and can occur as a result of irregular, asymmetric movement in patterns and postures and changes in muscle strength, tone, or length. Pain-related to spasticity can include muscle aching, cramping, pulling, or stiffness. These symptoms are often worse at night or early in the morning [7,8]. Several studies reported that central neuropathic (dysesthetic) extremity pain is the most common type of pain associated with MS, followed by back pain, headache, and painful tonic spasms [9-11]. Although its physical, psychological, and social impact is not well-understood [11].

There is only one Portuguese study with a small sample size that shows data on prevalence of pain [12]. This study was designed to evaluate the pain experience among patients with multiple sclerosis in one of the biggest Portuguese health institutions, by describing its prevalence, characteristics, analgesic treatment, and its efficacy, and also the impact of pain on quality of life.

## Materials and methods

This cross-sectional observational study took place in an MS clinic at Centro Hospitalar Universitário de Lisboa Central (CHULC), a tertiary hospital in Lisbon. All patients older than 18 years old were considered eligible. Exclusion criteria were inability to read or write or to consent to voluntary participation.

Data was collected between December 2019 and March 2021 from a structured telephone inquiry, applying two questionnaires, the Brief Pain Inventory and The McGill Pain Questionnaire (MPQ), to evaluate the prevalence of pain and its impact on quality of life (QoL). Clinical records were also consulted to obtain data on disease duration, year of diagnosis, MS type, Expanded Disability Status Scale (EDSS) score. The project was approved by the CHULC Ethics Committee with the approval number AGFC/79/2019 and all patients gave informed consent.

Pain evaluation and questionnaires

Brief Pain Inventory

The BPI is a simple and short questionnaire composed of 15 items aiming to assess two scales: pain intensity and pain interference [13]. The pain intensity scale contains four pain intensity items of maximum, minimum, on average, and right now, measured with an 11-point numeric rating scale, ranging from 0 (no pain) to 10 (the worst pain possible). The pain interference scale is composed of seven items of patient’s pain-related interference regarding general activities, mood, walking ability, normal work, relations with other people, sleep, and enjoyment of life, measured also with an 11-point scale, ranging from 0 (no interference) to 10 (extreme interference). BPI is translated in 10 different languages, including a Portuguese version, and has been shown to have excellent psychometric properties [12]. Therefore, it is an instrument recommended for clinical and epidemiological research and highly consensual on the guidelines for pain assessment [14,15].

Long-Version of the McGill Pain Questionnaire

The McGill Pain Questionnaire (MPQ) measures the sensory, affective, evaluative, and miscellaneous components of pain [16]. It was developed by Dr. Melzack and allows a very comprehensive analysis of several aspects of patient’s pain which are not analyzed by the BPI [14,15]. The questionnaire allows individuals to describe the quality and intensity of their pain by using 78 adjectives in 20 different sections. In addition to the list of pain descriptors, the questionnaire also allows to perceive the spatial distribution of the pain, contain words that describe temporal properties of pain, and descriptors of the overall present pain intensity (PPI). The PPI is recorded on a 5-point scale, in which each number is associated with the following words: (1) mild, (2) discomforting, (3) distressing, (4) horrible, and (5) excruciating.

Statistical analysis

The information gathered was entered into an excel sheet. An exploratory analysis was carried out for all variables. Categorical data were expressed as frequencies and percentages, and continuous variables as mean or median and SD. Univariable analysis was carried out using Student’s t-test and nonparametric chi-square test as appropriate. Pearson’s correlation was used to investigate association between pain intensity and disease duration. Results were considered significant when p was less than 0.05. Confidence intervals are presented when appropriate (95% CI). Statistical analysis was carried out using the IBM SPSS Statistics, version 27.0 (Armonk, NY: IBM Corp.).

## Results

Description of the sample

Four hundred phone calls were made, and only 305 patients answered the questionnaire and consented to data collection. Of the remaining 95 patients, 40 patients did not consent to participate in the study, 45 patients did not attend, and 10 were dependent, so only the caregiver could provide data and consent. These last 10 patients were also excluded from the sample. The patient’s demographic data and disease characteristics are presented in Table 1. The sample was predominantly female (64.6%) with a mean age of 44.27 years (SD: 11.58) and well-educated (51.8% attended at least 12 years of school).

**Table 1:** Demographic and disease characteristics of sample (n=305). EDSS: Expanded Disability Status Scale

Variable		n (%)
Gender	Female	197 (64.6%)
	Male	108 (35.4%)
Scholarity	≤ 4 years	9 (3%)
	5 to 9 years	38 (12.4%)
	10 to 12 years	100 (32.8%)
	> 12 years	158 (51.8%)
EDSS classification	Mild (0-4.0)	257 (84.3%)
	Moderate (4.5-6.0)	30 (9.8%)
	Severe (6.5-9.5)	18 (5.9%)
Disease subtype	Relapsing-remitting	270 (88.5%)
	Primary progressive	21 (6.9%)
	Secondary progressive	14 (4.6%)
	Clinically isolated syndrome	0 (0%)

Concerning multiple sclerosis, the meantime since initial diagnosis was 9.72 years (SD: 7.63). The mean EDSS score was 2.18 (SD: 1.99). Two hundred and seventy patients (88.5%) presented with relapsing-remitting subtype of disease, 21 (6.9%) with primary progressive, and 14 (4.6%) with secondary progressive subtype.

Pain prevalence and characteristics

The prevalence of pain in this population was 40.65%. One hundred eighty-one did not experience any pain. One hundred twenty-four experienced pain a week before and answered the Brief Pain Inventory (BPI) questionnaire and the Portuguese version of McGill pain questionnaire (MPQ).

Regarding pain location, only 54 (43.5%) patients experienced pain in a single point, against 49 (39.5%), 14 (11.3%), and seven (5.6%) that experienced pain at two, three, or four locations, respectively. The most frequent sites were lower limb pain (68.5%), back pain (40.3%), upper limb (36.3%), and head pain (16.1%) (Table 2).

**Table 2:** Anatomical regions where patients experienced the most pain. *The total percentage of pain is >100 due to the fact that some patients experienced pain in more than one anatomical region.

Location	Percent with pain*
Lower extremity	68.5
Lower back	40.3
Upper limb	36.3
Head	16.1
Right or left body	12
Hemifacial	5
Ocular	25

Using BPI, pain severity was calculated through mean value and standard deviation from a 10-point scale. Pain severity calculated was 6.25 (SD: 2.02) for maximum, 1.94 (SD: 2.04) for minimum, 4.47 (SD: 2.71) for mean, and 3.29 (SD: 2.74) for current pain intensity. At MPQ, almost 97% of the patients described their pain with words of the four dimensions, proposed by the questionnaire. One hundred twenty-four patients used at least one word of the sensitive category, 112 a word of the affective category, 104 a word of the evaluative category, and 111 one word of the miscellaneous group. Table 3 resumes the scores obtained with MPQ.

**Table 3:** Main words chosen by category in MPQ and pain index. MPQ: McGill pain questionnaire

	Main words (%)	Pain index (SD)
Sensory	Tender (45.2%), boring (44.4%), tingling (41.9%), throbbing (40.3%), hot (29.8%)	17.4 (7.34)
Affective	Tiring (60.5%), sickening (33.0%)	4.24 (3.04)
Evaluative	Miserable (37.1%)	2.40 (1.12)
Miscellaneous	Nagging (35.5%), radiating (24.2%)	5.01 (1.42)
Total		29.03 (5.92)

The most selected descriptors were tender (45.2%), boring (44.4%), tingling (41.9%), miserable (37.1%), and nagging (35.5%). The calculated pain index (standard deviation) for the sensitive dimension was 17.4 (7.34), while for the affective dimension a value of 4.24 (3.04) was obtained, and the total pain index was 29.03 (5.92). Considering current pain, and a 0 to 6 points scale, the mean intensity was 1.81 (0.9), below the middle point, and so non-significant. No association between pain intensity and disease duration was statistically significant (p=0.21). Concerning pain patterns, 46.8% of the patients defined it as rhythmic, periodic, or intermittent, while 36.3% recognized their pain as being continuous, steady, or constant.

Pain and interference with daily function

Pain interference with daily function is scored 4.77 (SD: 3.10). The mean effect of pain over general activity was 5.83 (SD: 2.728), humor 5.31 (SD: 2.948), walking 4.72 (SD: 3.360), regular work 5.65 (SD: 2.981), relationships 4.11 (SD: 3.323), sleep 4.11 (SD: 3.323), and pleasure of life 3.68 (SD: 3.070).

Pain management

Eighty-four patients (67.7%) who experienced pain were under analgesic treatment. Among patients who take one drug (64.3%), NSAIDs were the most used followed by gabapentinoids and acetaminophen. Seventeen patients (20.2%) were under treatment with two drugs, eleven (13%) with three drugs, and four (4.7%) with more than three drugs. The mean pain relief analgesic treatment was 54%. No association between a specific type of treatment and pain relief was found.

Comparisons of persons with and persons without pain

Regarding both cohorts, patients without pain and with pain, no statistically significant differences were found respect to gender (chi-square test, p=0.15), EDSS score (chi-square test, p=0.870), or mean time of disease (t-test, p=0.956). However, the difference of ages between samples is statistically significant (t-test, p=0.004), with older patients in the pain group. This information is detailed in Tables 4, 5.

**Table 4:** Comparison of the socio-demographic characteristics of the two cohorts. Data are presented as mean (SD) or number (%). *Student's t-test. **Chi-square test.

Variable		Patients without pain (n=181)	Patients with pain (n=124)	p-Value
Age (years)		42.75 (11.52)	46.60 (11.34)	0.004*
Gender	Female	111 (61.3%)	86 (69.4%)	0.150**
	Male	70 (38.7%)	38 (30.6%)	
Scholarity	≤ 4 years	2 (1.1%)	7 (5.6%)	0.08**
	5 to 9 years	19 (10.5%)	19 (15.3%)	
	10 to 12 years	55 (30.4%)	45 (46.3%)	
	> 12 years	105 (58.1%)	53 (42.7%)	

**Table 5:** Comparison of clinical characteristics of the two cohorts. *Independent samples Student's t-test. **Chi-square-test. EDSS: Expanded Disability Status Scale

Variable		Patients without pain (n=181)	Patients with pain (n=124)	p-Value
Time of disease (years)		10.41 (7.80)	10.36 (8.26)	0.956*
Time since diagnosis (years)		9.86 (7.63)	9.52 (7.67)	0.709*
EDSS		2.08 (2.00)	2.32 (1.99)	0.312**
Subtype	Clinically isolated syndrome	0 (0%)	0 (0%)	--
	Relapsing-remitting	162 (89.5%)	108 (87.1%)	--
	Secondary progressive	8 (4.4%)	6 (4.8%)	--
	Primary-progressive	11 (6.1%)	10 (8.1%)	--

## Discussion

According to 2016 data, it was estimated that there were between 56,000 and 8000 patients with MS in Portugal [17]. This cross-sectional observational study took place in MS clinic at Centro Hospitalar Universitário de Lisboa Central (CHULC), a tertiary hospital in Lisbon, which treats around 1500 MS patients a year.

The socio-demographic data of our sample are similar to other studies, with the majority of patients being female and suffering from the clinical subtype of outbreak-remission of the disease [4,6]. The high level of education found in the sample of patients was an interesting finding and may have had an influence on the high number of patients who managed to complete the applied pain questionnaires (BPI and MPQ).

As previously mentioned, 40.65% of patients experienced pain during the week prior to the questionnaire, which demonstrates a prevalence of pain similar to other populations studied [1,2,7,12]. The risk factors reported in other studies to be associated with a greater likelihood of pain in patients with MS include older age, longer disease duration, and greater disease severity [7,9]. In our study, pain was associated with increased age only. The fact that our study does not demonstrate a relationship with duration of illness or degree of functional impairment could be related with a low EDSS average. The average pain intensity in patients with MS was found to be moderate. Although, according to Daut et al., pain intensity or interference in QoL can be considered meaningful above the median point 5 of the BPI scales [18]. Only the maximum pain intensity reported by patients was considered significant in our study.

Regarding pain location, our studies are consistent with others [1,19]. Regarding the sensorial component of The McGill Pain Questionnaire, we found that 41.9%, 40.3%, and 29.8% choose tingling, throbbing, and hot as pain descriptors, respectively. However, the most chosen descriptors were tender (45.2%) and boring (44.4%). As previously mentioned, dysesthetic central extremity pain is the most common type of pain associated with MS, being described as a burning, throbbing, stabbing, or aching pain in the legs and feet bilaterally and is often worse at night [4-9]. The data obtained in the present study are not sufficient to evaluate the prevalence of central vs nociceptive pain, such categorization would require a more detailed questionnaire and eventually a medical assessment.

Regarding the management of pain, a greater number of patients should be under treatment. Perhaps the effectiveness of AINE's is related to nociceptive component of pain, which is not negligible, taking into account the McGill Pain Questionnaire results [20,21]. Pain significantly interfered with affective (mood) and activity subdimension (general activity and regular work). The impact at the physical level was greater than the emotional one, which may explain the lower impact at the level of social and affective relationships. These findings may be related to a higher educational level and a greater ability to adapt to adversity. However, the impact at the physical level should not be overlooked as it can lead to poor quality of life, absence from work, increased disability level, mental health deterioration, and financial problems [22,23].

Finally, the greatest difficulty found in the studies is the lack of a standardized questionnaire to assess the prevalence of pain and distinguish the different types of pain in this population. It would be very interesting to develop new questionnaires aimed at this population that would allow the prevalence of pain to be measured in a more uniform way and a valid distinction between neuropathic pain and nociceptive pain [24].

## Conclusions

With our cross-sectional survey using two validated questionnaires, we found that pain is an important symptom in patients with MS with several implications for patient satisfaction and quality of life. The maximum intensity of pain felt by patients is significant and only 67.7% of patients are under analgesic treatment with mean pain relief of 54. NSAIDs were the most drug used followed by gabapentinoids and acetaminophen for management of pain. Since pain remains a frequent and often overlooked problem, medical community must continue to study this population in order to improve the approach to pain in these patients and improve quality of life.
